# Human ecological and social determinants of dental caries among the Xavante Indigenous people in Central Brazil

**DOI:** 10.1371/journal.pone.0208312

**Published:** 2018-12-19

**Authors:** Rui Arantes, James R. Welch, Felipe Guimarães Tavares, Aline Alves Ferreira, Mario Vianna Vettore, Carlos E. A. Coimbra

**Affiliations:** 1 Fundação Oswaldo Cruz Mato Grosso do Sul, Campo Grande, MS, Brazil; 2 Escola Nacional de Saúde Pública, Fundação Oswaldo Cruz, Rio de Janeiro, RJ, Brazil; 3 Escola de Enfermagem Aurora de Afonso Costa, Universidade Federal Fluminense, Niterói, RJ, Brazil; 4 Instituto de Nutrição Josué de Castro, Universidade Federal do Rio de Janeiro, Rio de Janeiro, RJ, Brazil; 5 School of Clinical Dentistry, The University of Sheffield, Sheffield, United Kingdom; University of Queensland, AUSTRALIA

## Abstract

This community study evaluates complex interactions between macro and micro determinants of oral health in a local Indigenous population based on a theoretical framework of demographic, economic, and healthcare transformation over the last half century. The study population included all residents of eight Xavante villages in Central Brazil. Our hypothetical model posited multiple direct and indirect associations between dental caries and village groups with differentiated territorial and oral care histories, as well as household socioeconomic indicators and food acquisition patterns, individual sociodemographic characteristics, use of dental health services, and oral hygiene practices. Structural equation modelling methods were used to evaluate direct and indirect associations linking exogenous factors and dental caries. Results include 18 direct and 14 indirect statistically significant pathways between determinant variables and dental caries. Significant links with dental caries were shown for socioeconomic indicators, oral healthcare variables, household food acquisition patterns, sex, and age. These findings suggest that the oral health of Xavante residents in the villages studied is associated with determinant factors of different epidemiological and historical scales. The specific historical frame of territorial circumscription and demographic crisis followed by rapid population increase since the 1970s should be considered a cause-of-cause determinant of the economic, healthcare, and sociodemographic profile contributing to oral health among the Xavante. Considering the limitations of cross-sectional studies, our findings underline the importance for oral health determination of historical currents affecting minority ethnic groups within national societies.

## Introduction

Dental caries continues to be one of the most prevalent diseases and a major public health problem worldwide despite the availability of highly effective basic prevention measures. This is especially the case among populations who are poorer, socially excluded, and lack adequate access to health services [1–3]. In Brazil’s national population, a major oral health transition accompanying improved access to preventive oral health services occurred in recent decades, with pronounced but unevenly distributed reductions in the prevalence of dental caries in all age groups [4]. From 1980 to 2003, decayed, missing, and filled teeth (DMFT) index values fell by 61.7% overall. However, approximately 60% of the dental caries disease burden is concentrated in just 20% of the national population [3,5,6]. Disadvantaged social groups and regions with low human and social development indicators, inadequate primary care services, and low or non-existent access to fluoride were predominantly affected.

For much of Brazil’s Indigenous population, industrialized and ultraprocessed foods are now part of everyday diets, overwhelmingly influencing the course of health and nutritional transitions, contributing to the onset of chronic diseases, and promoting rapid increase in caries and other oral health disorders [7,8]. A growing literature about oral health status of different contemporary Indigenous ethnic populations in Brazil converge with respect to at least two common findings—very high levels of dental caries in children and younger adults and edentulism among middle-aged adults and the elderly [9,10].

Evidence suggests these unfavorable oral health changes result from interplay between dietary changes and insufficient access to preventive resources and healthcare services [10,11]. The circumstances of oral and dietary health transition among Indigenous peoples in Brazil are similar to other world regions in which subsistence and foodways transformations are driven by economic and sociocultural changes accompanying involvement with non-Indigenous national societies [12]. Accordingly, it is likely that the underlying causes of oral health inequalities include recent patterns of migration and urbanization, territorial loss and environmental degradation, social and political exclusion, and postcolonial structural racism [13].

Since 1990, our research group has conducted ongoing anthropological and health investigations among the Indigenous Xavante people residing in the Pimentel Barbosa Indigenous Reserve, Central Brazil [14–21]. Building on these studies, in 2011 we undertook a survey addressing health and territoriality in all contemporary villages that were historically derived through division and migration from a single village with historical baseline data collected by James V. Neel and Francisco M. Salzano in the early 1960s [22]. This expansion of our research scope permitted comparative analyses between groups of villages with known histories of geographical and social differentiation over a period of about four decades. The present study sought to draw on this rich historical record to evaluate potential determinants of dental caries. Ultimately, the goal was to ascertain interactions between macro and micro determinants of oral health [23] in a local Indigenous population that has undergone rapid social, economic, and environmental transformation. Building on theoretical and analytical frameworks that are unique in the literature of oral health among Indigenous peoples in Brazil and elsewhere, this community study provides broad insights into how multiple scales and dimensions of determinants can affect emergent oral health disparities in historically marginalized ethnic minorities.

Our theoretical framework is anchored in three historical processes with high potential to have impacted oral health in the study villages. The first involves demographic and village dynamics since the population permanently settled within present reservation boundaries in the early 1970s. After decades of population decline and instability since contact was established with the Brazilian federal government in 1946, the Xavante of Pimentel Barbosa began to undergo rapid population growth, leading to numerous village divisions and relocations. This ongoing process reflects traditional aspects of Xavante social organization, as well as such extrinsic factors as proximity to highways, markets, and public services [24,25]. The second is recent economic transformation linked to increased involvement with the regional market economy and access to income through employment, social benefits, and development initiatives. These economic changes have complex consequences for the maintenance of traditional subsistence activities and other household food acquisition practices [19,26]. The third process involves improved access to oral health services, education, and preventive care through recent public and private initiatives [18].

We evaluated potential contributions of contemporary social, environmental, and biological circumstances produced through these three interrelated historical processes to the determination of oral health. Direct and indirect associations between dental caries and village groups with differentiated territorial and oral care histories were analyzed along with household socioeconomic indicators and food acquisition patterns, individual sociodemographic characteristics, use of dental health services, and oral hygiene practices. We tested the theoretical model using structural equation modelling methods, which permitted the creation of pathway diagrams identifying which determinant variables directly predicted or were indirectly linked via mediating variables to dental caries and intermediate outcomes.

## Methods

### Population and fieldwork

In 2011, the Xavante population resided in ten federally recognized Indigenous reserves located in northeastern Mato Grosso state, Central Brazil. The present study aimed to include all nine villages then located in the Pimentel Barbosa and Wedezé Indigenous reserves that were predominantly derived through natural increase and migration from residents of São Domingos (1950s) and subsequently Pimentel Barbosa village (1970s) [22]. Beginning in 1980, various subgroups left Pimentel Barbosa village to establish new villages elsewhere in these two adjacent reserves, some of which subsequently underwent further divisions.

The cross-sectional data reported here were collected in July and August 2011. Oral health data were sought for all individuals ≥ 5 years of age, among whom permanent teeth are most prevalent. No sample techniques were employed. A multidisciplinary research team including public health researchers with backgrounds in anthropology, nutrition, nursing, and dentistry participated in data collection. Interviews with questions used in the present study, shown in S1 Interview Questions, and clinical examinations were conducted at participants’ homes. Clinical examinations were conducted by five previously calibrated dentists to assess oral diseases including dental caries. Inter-examiner agreement (minimal Cohen’s kappa-value of 0.68) was substantial [27]. Participants’ dental caries in permanent teeth were observed under natural illumination using a ballpoint probe. Criteria proposed by the World Health Organization for oral health surveys were used to classify the tooth surfaces as decayed, missing, or filled [28].

Interview instruments were based on those used in the First National Survey of Indigenous People’s Health and Nutrition in Brazil [7], adapted to local circumstances and cultural specificities of the study villages and complemented with additional investigative topics, including oral health. Household interviews with heads of household or other knowledgeable adult residents addressed sanitation conditions, food acquisition patterns, and socioeconomic status. Individual interviews addressed education, reproductive and health history, access to health services, and health status. Individual interview questions related to oral health included dental services use and oral hygiene habits.

Investigated villages were assigned to three groups based on their distinct territorial histories of division and migration to create a socio-geographical indicator (“village group”). The first group included Pimentel Barbosa village, which was founded in 1970 and from which all other participating villages were historically derived. Etênhiritipá village, which split from Pimentel Barbosa village in 2006, was also included in the first group. This group was the farthest by road from nearby towns and located in an area of relatively undegraded cerrado vegetation [19,29]. Differently from the second and third village groups, this one benefitted from a privately funded oral health program undertaken by the lead author from 1999 to 2009, as described below [10,30,31]. The second group of villages included Caçula village, which separated from Pimentel Barbosa village in the early 1980s, and two smaller villages that separated from Caçula in the 2000s (Canoa and Wedezé). This group was in closest proximity to the Rio das Mortes, a major tributary of the Araguaia River, in an area of seasonally inundated grasslands previously occupied by large ranches. The third group included Tanguro village, which separated from Pimentel Barbosa village in the mid-1980s, and two smaller villages that separated from Tanguro in the 2000s (Asereré and Reata). This group was closest to the nearest small town, interstate highway, and currently operating ranches. To protect the confidentiality of villages and their residents, our analyses and interpretations do not present findings for individual villages.

Residents of the three village groups were largely genealogically related to one another, maintained frequent social contact, and participated in substantially similar subsistence and local market economies. All groups had access to primary and secondary education and primary healthcare services, which varied between villages in terms of proximity, facilities, and personnel. Beyond these basic similarities, the village groups’ differentiated historical trajectories, ecological settings, and access to market services and products are accompanied by demographic and economic distinctions with potential implications for foodways, socioeconomic status, and dental health [19]. As mentioned above, these potential similarities and differences between village groups figure importantly in the theoretical framework for our analytical model.

Villages were also assigned to two groups based on presence or absence of the privately funded oral health program from 1999 to 2009 [30,31]. This project, undertaken in Pimentel Barbosa and Etênhiritipá villages, incorporated community participation to develop an oral health education program, promote dental caries prevention, and provide clinical dental services. The educational aim was to encourage beneficial oral health interest and selfcare habits among children and adults. The preventive component was designed to ensure access to fluoride by furnishing toothpaste in combination with fluorotherapy. Indigenous assistants from each village were trained to apply topical fluoride and distribute oral hygiene material (dental floss, fluoride toothpaste, and toothbrushes). The project’s clinical component entailed providing dental services for pain alleviation, treatment of infections, atraumatic restorative treatment, non-recoverable tooth extraction, and basic periodontal therapy [10,30].

The presence of durable industrial goods in each household (henceforth “durable goods”) was assessed based on a nearly comprehensive reference list of 22 items developed in collaboration with community members. Household food acquisition patterns were assessed based upon an extensive list of foods previously developed in collaboration with Xavante interlocutors. This list included 44 items grouped into three categories: cultivated or raised foods (16 food items produced in villages and household gardens), forest foods (11 items acquired by gathering, fishing, and hunting), and industrialized or purchased foods (17 items acquired from supermarkets and other sources outside the Indigenous reserves). Interviewees were asked how frequently each food item tended to be consumed in the household. Response options were (1) “never or rarely,” (2) “sometimes or seasonally,” and (3) “frequently or every day.”

The study was approved by the Research Ethics Committee at the Escola Nacional de Saúde Pública, Fundação Oswaldo Cruz, and the Brazilian National Ethics Council in 2011. Attending to Brazilian legislation requiring that research with Indigenous populations respect culturally distinct community protocols for authorizing research, the study was presented at an open meeting in each village and, if approved, leaders signed a Collective Informed Consent Form on behalf of the community. Individual participants or their guardians could decline or withdraw participation at any time.

### Analyses

Prevalence analyses were planned before data collection began. Other analyses were planned after data were collected and systematized. Structural equation modelling was planned after evaluating data adequacy. No subsequent data-driven changes to analyses took place.

The outcome variable dental caries was estimated by calculating DMFS index scores (the sum of decayed, missing, and filled dental surfaces) for all analyzed participants.

The three village groups were assigned codes from 1 to 3 following the sequence of their initial separation from the “mother village”: village group 1 (Pimentel Barbosa and Etênhiritipá), village group 2 (Caçula, Canoa and Wedezé), and village group 3 (Tanguro, Asereré and Reata).

A socioeconomic indicator was calculated using principal component analysis of the quantities of durable goods reported for each household. Before applying this technique, the correlation matrix between the quantities of the 22 items was calculated. The Kaiser-Meyer-Olkin measure reached 0.64, which exceeds the minimum value (0.60) recommended for proceeding with principal component analysis. The first component had an eigenvalue of 4.32 and accounted for 21.6% of the total variability in the dataset. This first component, which was used in defining the socioeconomic indicator, was explained by television, DVD, parabolic antenna, digital camera, generator, and audio speaker. The household durable goods index was calculated as the sum of all the products of each item’s quantity and contribution. Households were then classified according to tertiles of the combined distribution. Households with higher scores and in higher tertiles were considered to have higher socioeconomic status.

Household food acquisition scores for each of three categories (cultivated or raised foods, forest foods, and industrialized or purchased foods) were calculated as the sum of response values for all food items (1 to 3 points per food item) and treated analytically as continuous variables. As a household score, these indicators were intended to reflect patterns involving domestic food economies, not individual nutritional consumption. Thus, higher scores indicate greater diversity and/or frequency of food items acquired by households throughout the year. Per capita monthly income and age were treated as continuous variables. The variables access to oral health program (no or yes), toothbrushing frequency (never, sometimes, once per day, or twice or more per day), and use of dental services (never, more than one year prior, or during the prior year) were treated as ordinal.

Distributions were compared using Wilcox tests for means with standard deviation (SD) and chi-square test for prevalence ratios with confidence intervals (CI) (α ≤ 0.05). For consistency with the modelling technique described below, all means, including household variables, were calculated using individuals as the unit of analysis (denominator).

**Fig 1:**
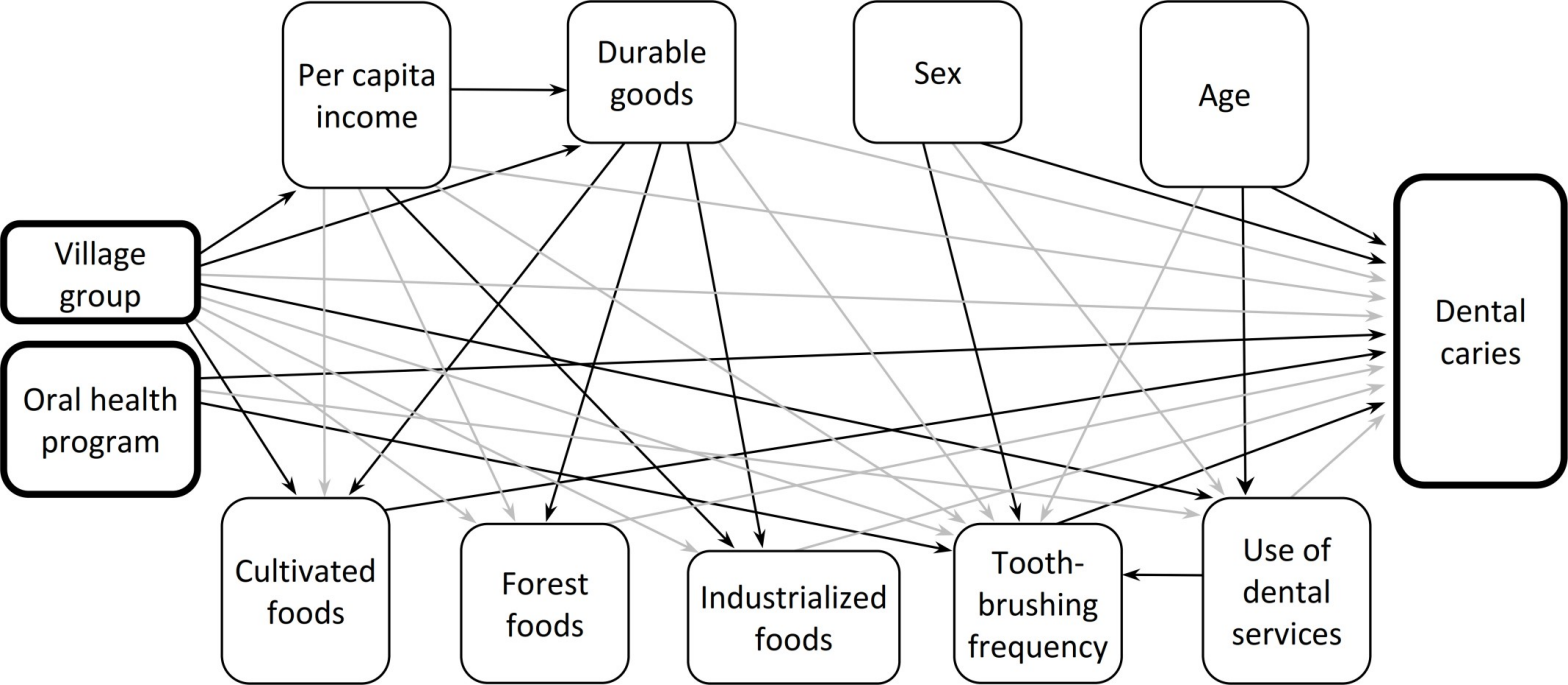
Hypothesized theoretical and parsimonious models of historical and socioeconomic determinants, use of dental services, frequency of toothbrushing, and dental caries among the Xavante indigenous population, Pimentel Barbosa and Wedezé Indigenous reserves, Brazil, 2011. Nonsignificant relationships removed before testing the parsimonious model indicated with grey arrows.

Structural equation modelling tested direct and indirect relationships between analyzed variables and the primary outcome variable, dental caries, according to the proposed theoretical model shown in Fig 1. In the theoretical model, the variables village group and access to oral health program were treated as exogenous (independent variables). This model hypothesized that village group would directly predict socioeconomic indicators, household foods acquisition, use of dental services, toothbrushing frequency, and dental caries. Access to the oral health program was hypothesized to predict use of dental services, toothbrushing frequency, and dental caries. It was also hypothesized that household foods acquisition, use of dental services, and toothbrushing frequency would predict dental caries. Age, sex, use of dental services, and toothbrushing frequency were furthermore hypothesized to predict dental caries. Indirect effects of village group on dental caries via socioeconomic indicators, household foods acquisition, use of dental services, and toothbrushing frequency were also hypothesized. Finally, the oral health program and demographic characteristics were hypothesized to predict indirectly dental caries via use of dental services and toothbrushing frequency.

The software program AMOS 22.0 was used to estimate total effects, which represents direct links between pairs of variables and indirect effects involving mediating links (e.g., oral health program linked to dental caries via toothbrushing frequency). Bias-corrected bootstrap CI were used to assess mediation by analyzing the statistical significance of indirect effects [32].

The initial hypothesized full model was estimated and the adequacy of the model fit was assessed according to five a priori criteria: the chi-square test (χ^2^/df ratio < 3.0), standardized root mean square residual (SRMR < 0.08), root-mean square error of approximation (RMSEA) with 90% CI ≤ 0.06, goodness of fit (GFI ≥ 0.95), and comparative fit indices (CFI ≥ 0.95) [33]. Non-significant direct paths between variables were removed to obtain a statistically parsimonious model, which was then re-estimated according to the same five criteria. Nine hundred bootstrap samples were re-sampled from the original data set to derive less biased standard errors and 95% CI bootstrap percentiles. The hypothetical and parsimonious models were then compared [Δχ^2^ (20)] to verify that elimination of pathways between variables were not statistically significant for the model. Standardized beta coefficients (β) for mediated (indirect) paths in the parsimonious model were calculated using the product of standardized beta coefficients for connecting direct paths.

The structural equation modelling technique is especially useful and well suited to assessing simultaneously the direct and mediated pathways between determinants and the outcome variable within a complex theoretical model. However, it is less effective for distinguishing the relative contributions or “importance” of distinct pathways, especially those involving one or more mediating variables. This methodological restriction is outweighed by its benefits for mapping the complex kinds of relationships addressed by our central objective, to evaluate diverse and potentially interrelated oral health determinants framed by a nuanced historical reading of oral health transition.

## Results

### Participant population and summary statistics

Of the nine historically related villages located in the Pimentel Barbosa and Wedezé Indigenous reserves in July 2011, eight participated in the study. The total resident population of participating villages was 1,337, of which 77.4% (1,035 individuals) were ≥ 5 years of age. The participation rate was 95.5% (988 individuals) and the final analyzed sample included 78.4% of participants (775 individuals). Nonparticipation and exclusion from analyses were due to physical disabilities (9 individuals), absence during fieldwork (28 individuals), declined participation (10 individuals), and missing data for one or more variables required for analyses (213 individuals). As shown in Tables 1 and 2, nonsignificant differences were observed between village groups in mean age (overall mean = 20.6 years; SD = 16.9) and proportion of females (overall proportion = 52.4%).

**Table 1:** Mean values for participants’ demographic, socioeconomic, and oral health variables, Pimentel Barbosa and Wedezé Indigenous reserves, Brazil, 2011.

		Mean (SD)			
Variable^a^	All villages	Village group 1	Village group 2	Village group 3	p-value^b^
Demographics					
Age (years)	20.6 (16.9)	19.7 (15.4)	22.7 (19.3)	20.3 (17.1)	0.822
Household socioeconomic characteristics					
Number of residents	15.8 (6.3)	16.4 (6.0)	16.5 (6.4)	12.3 (5.9)	< 0.001
Per capita income (R$)	96.52 (62.2)	103.23 (65.1)	86.24 (56.2)	92.60 (60.7)	0.001
Durable goods value	0.01 (0.1)	-0.1 (0.9)	0.1 (1.2)	0.1 (0.8)	0.108
Cultivated foods score	27.7 (3.4)	27.4 (2.9)	28.1 (4.5)	28.3 (2.7)	0.045
Forest foods score	21.2 (2.4)	21.0 (2.3)	22.7 (2.1)	19.5 (1.9)	< 0.001
Industrialized foods score	37.1 (3.3)	37.1 (2.9)	36.9 (3.8)	37.6 (3.8)	0.013
Oral health measures					
DMFS	17.2 (28.1)	14.0 (25.1)	21.4 (31.0)	20.4 (30.7)	0.003
Decayed surfaces	6.9 (13.5)	4.7 (10.6)	9.3 (16.1)	10.1 (15.8)	< 0.001
Missing surfaces	10.0 (20.5)	8.9 (19.3)	11.8 (21.9)	10.2 (22.1)	0.109
Filled surfaces	0.3 (1.4)	0.4 (1.3)	0.2 (1.9)	0.1 (0.4)	< 0.001
Number of teeth	22.1 (9.9)	21.5 (10.3)	22.5 (9.7)	22.1 (10.1)	0.368

α ≤ 0.05

^a^ all means, including household characteristics, calculated using individuals as the unit of analysis

^b^ Kruskal-Wallis test; DMFS decayed, missing and filled surfaces index; SD Standard deviation.

**Table 2:** Distribution of demographic and oral health indicators, Pimentel Barbosa and Wedezé Indigenous reserves, Brazil, 2011.

	All villages	Village group 1	Village group 2	Village group 3	
Variable	n (%) 95% CI	n (%) 95% CI	n (%) 95% CI	n (%) 95% CI	p-value^a^
Demographics					
Sex					0.183
Male	369 (47.6) 44.1–51.1	214 (50.6) 45.8–55.4	100 (43.5) 37.0–49.9	55 (45.1) 36.1–54.0	
Female	406 (52.4) 48.9–55.9	209 (49.4) 44.6–54.2	130 (56.5) 50.1–63.0	67 (54.9) 46.0–63.9	
Oral health measures					
Use of dental services					< 0.001
Never visited a dentist	366 (47.2) 43.7–50.8	165 (39.0) 34.3–43.7	115 (50.0) 43.5–56.5	86 (70.5) 62.3–78.7	
Last dental visit > 1 year prior	342 (44.1) 40.6–47.6	212 (50.1) 45.3–54.9	103 (44.8) 38.3–51.3	27 (22.1) 14.7–29.6	
Last dental visit during the prior year	67 (8.6) 6.7–10.6	46 (10.9) 7.9–13.9	12 (5.2) 2.3–8.1	9 (7.4) 2.7–12.1	
Toothbrushing frequency					< 0.001
Not usually	265 (34.2) 30.9–37.5	162 (38.3) 33.7–43.0	70 (30.4) 24.4–36.4	33 (27.0) 19.1–35.0	
Sometimes, when remembered	70 (9.0) 7.0–11.1	55 (13.0) 9.8–16.2	5 (2.2) 0.2–4.1	10 (8.2) 3.3–13.1	
Once per day	148 (19.1) 16.3–21.9	65 (15.4) 11.9–18.8	46 (20.0) 14.8–25.2	37 (30.3) 22.1–38.6	
Two or more times per day	292 (37.7) 34.3–41.1	141 (33.3) 28.8–37.8	109 (47.4) 40.9–53.9	42 (34.5) 25.9–43.0	

α ≤ 0.05

^a^ Chi-square test; CI Confidence interval; SD Standard deviation.

The mean per capita monthly household income varied significantly between village groups (p = 0.001), ranging from R$ 86.24 in village group 2 to R$ 103.23 in village group 1 (overall mean = R$ 96.52, equivalent to approximately US$ 39.40 at the time of data collection; SD = 62.2). Significant differences were observed between village groups with respect to cultivated foods (p = 0.045), forest foods (p < 0.001), and industrialized foods (p = 0.013) scores. The number of decayed, missing, and filled surfaces (DMFS) ranged from 0 to 129. The mean DMFS differed significantly between village groups (p = 0.003), ranging from 14.0 in village group 1 to 21.4 in group 2 (overall mean = 17.2; SD = 28.0). Differences were also observed between village groups for mean number of decayed surfaces (from 4.7 in group 1 to 10.1 in group 2; p < 0.001) and mean number of filled surfaces (from 0.1 in group 3 to 0.4 in group 1; p < 0.001). Differences between means were not significant for number of missing surfaces (overall mean = 10.0; SD = 20.5) and number of teeth (overall mean = 22.1; SD = 9.9).

Dental services use differed significantly (p < 0.001) between village groups. Whereas 10.9% of residents in village group 1 visited a dentist in the previous year, just 5.2% and 7.4% had done so in village groups 2 and 3, respectively. The proportions of individuals who had never visited a dentist were 39.0% (village group 1), 50.0% (group 2), and 70.5% (group 3). Toothbrushing frequency also differed (p < 0.001), with those reporting brushing at least once per day ranging from 48.7% in village group 1 to 64.8% in group 3.

### Structural equation model

**Fig 2:**
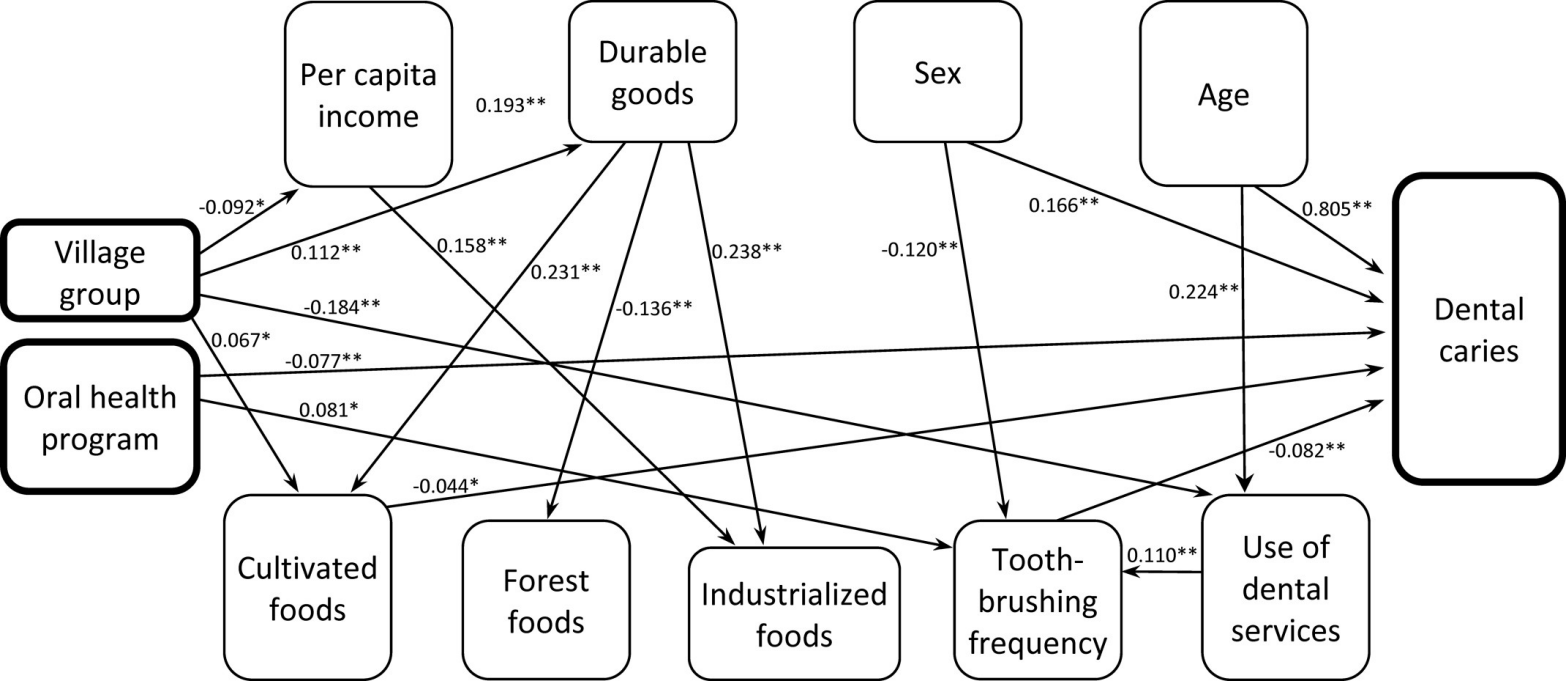
Significant direct effects (bootstrapped standardized estimates) in the final statistically parsimonious model, Pimentel Barbosa and Wedezé Indigenous reserves, Brazil, 2011. Direct effects indicated with solid arrows and corresponding p-values. *p < 0.05; **p < 0.01. Error terms and covariances omitted for ease of interpretation. See Table 3 for bias-corrected bootstrapped standardized estimates with confidence intervals.

**Fig 3:**
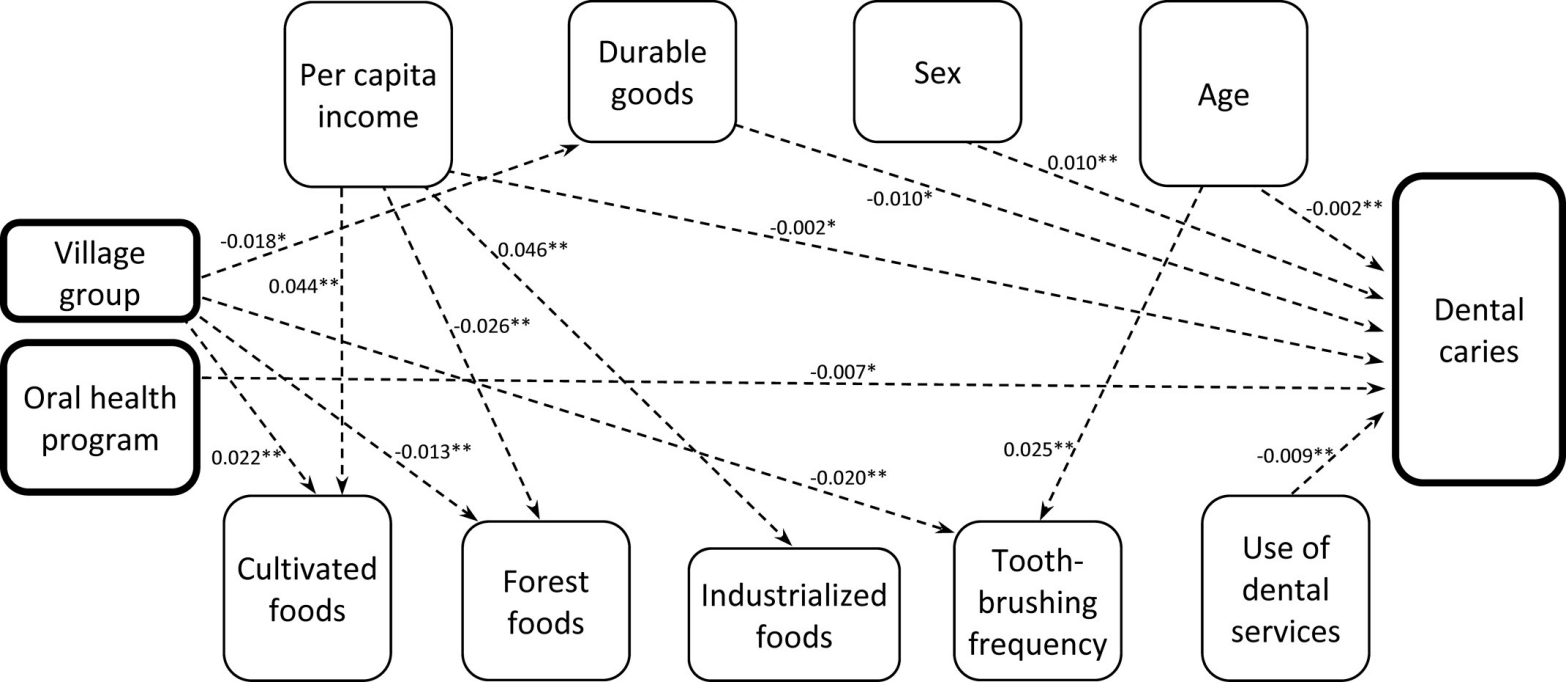
Significant indirect effects (bootstrapped standardized estimates) in the final statistically parsimonious model, Pimentel Barbosa and Wedezé Indigenous reserves, Brazil, 2011. Indirect effects indicated with dashed arrows and corresponding p-values. *p < 0.05; **p < 0.01. Error terms and covariances omitted for ease of interpretation. See Table 3 for bias-corrected bootstrapped standardized estimates with confidence intervals.

The hypothesized theoretical model (χ^2^/df ratio = 1.659, SRMR = 0.022, RMSEA = 0.029, GFI = 0.993, and CFI = 0.995) and the parsimonious model (χ^2^/df ratio = 1.249, SRMR = 0.024, RMSEA = 0.018, GFI = 0.991, and CFI = 0.997) presented in Fig 1 showed acceptable fit to the data and met all a priori criteria. The difference between the hypothetical and parsimonious models was not significant [Δχ^2^ (20) = 10.603]. In the final parsimonious model, DMFS accounted for 69.0% of overall variance. Bias-corrected bootstrapped standardized estimates with CI are presented in Table 3. Direct and indirect paths with standardized beta coefficients in the parsimonious model are presented in Figs 2 and 3, respectively.

**Table 3:** Significant bias-corrected bootstrapped standardized direct and indirect estimates with confidence intervals, Pimentel Barbosa and Wedezé Indigenous reserves, Brazil, 2011.

	β	Bootstrap SE	Bias-corrected 95% CI
Direct effects			
Village group–per capita income	-0.092	0.036	-0.163 / -0.022*
Village group–durable goods	0.112	0.033	0.050 / 0.176**
Village group–use of dental services	-0.184	0.035	-0.251 / -0.110**
Village group–cultivated foods	0.067	0.029	0.011 / 0.124*
Oral health program–toothbrushing frequency	0.081	0.037	0.007 / 0.151*
Oral health program–dental caries	-0.077	0.021	-0.119 / -0.038**
Per capita income–durable goods	0.193	0.041	0.105 / 0.264**
Per capita income–industrialized foods	0.158	0.032	0.097 / 0.223**
Durable goods–cultivated foods	0.231	0.047	0.133 / 0.323**
Durable goods–forest foods	-0.136	0.031	-0.199 / -0.076**
Durable goods–industrialized foods	0.238	0.033	0.171 / 0.305**
Cultivated foods–dental caries	-0.044	0.022	-0.089 / -0.002*
Sex–toothbrushing frequency	-0.120	0.035	-0.188 / -0.051**
Sex–dental caries	0.166	0.019	0.128 / 0.202**
Age–use of dental services	0.224	0.034	0.154 / 0.288**
Age–dental caries	0.805	0.018	0.768 / 0.836**
Use of dental services–toothbrushing frequency	0.110	0.035	0.036 / 0.180**
Toothbrushing frequency–dental caries	-0.082	0.021	-0.123 / -0.040**
Indirect effects			
Village group–durable goods	-0.018	0.009	-0.039 / -0.003*
Village group–forest foods	-0.013	0.005	-0.025 / -0.004**
Village group–cultivated foods	0.022	0.009	0.007 / 0.040**
Village group–toothbrushing frequency	-0.020	0.008	-0.035 / -0.006**
Oral health program–dental caries	-0.007	0.001	-0.014 / -0.001*
Per capita income–cultivated foods	0.044	0.013	0.021 / 0.070**
Per capita income–forest foods	-0.026	0.008	-0.044 / -0.011**
Per capita income–industrialized foods	0.046	0.011	0.023 / 0.068**
Per capita income–dental caries	-0.002	0.001	-0.005 / -0.001*
Durable goods–dental caries	-0.010	0.006	-0.024 / -0.001*
Sex–dental caries	0.010	0.004	0.003 / 0.019**
Use of dental services–dental caries	-0.009	0.004	-0.017 / -0.002**
Age–toothbrushing frequency	0.025	0.009	0.008 / 0.045**
Age–dental caries	-0.002	0.001	-0.004 / -0.001**

* p < 0.05

** p < 0.01

β standardized coefficient; CI Confidence interval; SE Standard error.

### Significant pathways of caries determination

The sequence by which village groups separated from a single “mother” village since the 1980s was directly linked to lower income (β = -0.092), higher durable goods indicator values (β = 0.112), and higher cultivated foods scores (β = 0.067). Higher income was directly linked to greater durable goods indicator values (β = 0.193). The village group sequence was also indirectly linked with lower durable goods values via lower per capita income (β = -0.018). Fewer dental caries was indirectly predicted by (a) higher per capita income via higher durable goods values and cultivated foods scores (β = -0.002) and (b) higher durable goods values via higher cultivated foods scores (β = -0.010).

The village group sequence was indirectly linked with (a) higher cultivated foods scores via higher durable goods values (β = 0.022) and (b) lower forest foods scores via higher durable goods values (β = -0.013). Higher income directly predicted higher industrialized foods scores (β = 0.158). Higher durable goods values were directly linked to higher cultivated foods scores (β = 0.231), lower forest foods scores (β = -0.136), and higher industrialized foods scores (β = 0.238). Higher durable goods values also mediated indirect links from higher income to all three foods scores (cultivated, forest, and industrialized). Higher cultivated foods scores directly predicted lower dental caries (β = -0.044).

Greater use of dental services was directly linked to increased toothbrushing frequency (β = 0.110) and indirectly predicted fewer dental caries via greater frequency of toothbrushing (β = -0.009). The village group sequence was directly linked to less use of dental services (β = -0.184) and indirectly linked to lower toothbrushing frequency via less use of dental services (β = -0.020).

Presence of the private oral health program in some villages from 1999 to 2009 directly predicted more frequent toothbrushing (β = 0.081) and fewer dental caries (β = -0.077). This program also indirectly predicted fewer dental caries (β = -0.007) via higher toothbrushing frequency. More frequent toothbrushing directly predicted fewer dental caries (β = -0.082).

Female sex predicted more dental caries directly (β = 0.166) and indirectly via less frequent toothbrushing (β = 0.010). Being older was directly linked to greater use of dental services (β = 0.224) and more dental caries (β = 0.805). Greater age was also indirectly linked with (a) more frequent toothbrushing via use of dental services (β = 0.025) and (b) fewer dental caries via use of dental services and more frequent toothbrushing (β = -0.002).

## Discussion

### Oral health transition

Major subsistence and dietary changes with repercussions for demographic patterns, health processes, and perceptions of wellbeing mark the historical trajectories of human societies, especially since staple plant foods were domesticated in multiple world regions. The widespread Agricultural Revolution (ca. 10,000–12,000 BP) involved a switch in economic emphasis from hunting and gathering to agriculture that increased supply of dietary calories and contributed to circumstances favorable to population growth [34,35]. Human health consequences of this shift included dental caries emerging as a major endemic disease due to the high cariogenic potential of many cultivated foods (e.g., maize and manioc) [36].

In the Americas, bioarcheological studies have shown that adoption of primarily maize-derived carbohydrate diets was directly related to overall decline in oral health, with higher rates of dental caries, periodontal disease, and malocclusion in comparison with hunter-gatherers, who show very low frequencies of oral disease [37]. For example, evidence from ca. 5,000 BP of hunter-gatherer “Sambaqui people” in coastal areas of southern Brazil show their diets of predominantly mollusks, crustaceans, and fish was associated with nearly perfect oral health, including strong jaws and very few indications of caries, although dental arches were almost always very worn [38,39].

About 10,000 years later, the Industrial Revolution (ca. 1760 to 1840) involved a second global oral health transition as many human diets shifted in emphasis to processed foods with rapidly absorbed simple sugars, provoking changes in oral microbial flora and epidemic tooth decay and gum disease [36]. Notably, all of these historical oral health transitions were based in economic change. In the case of the Xavante, oral health change associated with increased emphasis on agriculture is very recent and comingled with uneven distribution of contemporary public and private oral health services, as well as selfcare patterns.

According to James V. Neel and Francisco M. Salzano, who conducted biomedical research in the Pimentel Barbosa Indigenous Reserve in the early 1960s, “[…] Xavante adults exhibited broad dental arches, almost perfectly aligned teeth, end-to-end bite, and extensive dental attrition. At the age of 18–20 years, the teeth were so worn out as to almost totally obliterate the cusp patterns, leaving flat chewing surfaces” [40] (p. 545). They explained these conditions as resulting from a diet based on hunting and collecting, complemented by limited agricultural foods from traditional gardens. The same research team also visited Simões Lopes, a Xavante village located in proximity to an evangelical mission, where they found caries in 67% of examined subjects. This marked difference between the two villages was explained by the fact that “[…] for some five years, the Simões Lopes Xavante have had access to sugar cane, whereas none was grown at São Domingos” [40] (p. 544).

The resident population of Pimentel Barbosa and Wedezé reserves experienced significant declines in oral health over the last half-century due to major changes in economic and dietary conditions [18]. The mean DMFT in the age group 20 to 34 rose from 0.7 in 1962 to 9.7 in 1997 in the Pimentel Barbosa and Etênhiritipá village population (prior to their division into separate villages) [10]. Among those 35 to 44 years old, the increase in DMFT over the same period was from 2.4 to 14.3. Comparing results from the present study for village group 1 with those of a study in the same population 7 years earlier [41], the overall mean DMFS among individuals ≥ 5 years increased by 13.8%. Although similar baseline data are not available for the other village groups analyzed in the present study, this finding suggests oral health decline is an acute and ongoing community health issue in the study population.

### Socioeconomic status and household food economies

Our results reveal significant differences in socioeconomic conditions (mean per capita monthly income) and household access to foods (cultivated, forest, and industrialized) between the three village groups. These contrasts signal what Coimbra and Santos [42] characterized in the early 1980s as emergent internal socioeconomic differentiation within historically egalitarian Indigenous communities. This process potentially contributes to health inequalities within and between villages in contemporary Indigenous societies, such as the significant differences observed between village groups in oral health (mean DMFS and mean number of decayed surfaces) and oral care (use of dental services and toothbrushing frequency). As we discuss below, these disparities between village groups involve complex interactions of determinants related to their distinct historical trajectories within local and national scenarios of demographic crisis and recovery, territorial circumscription, socioeconomic change, and transformations in access to healthcare.

Both measures of higher household socioeconomic status (per capita monthly income and durable goods values) predicted better oral health. A study conducted among Indigenous Terena, Guarani, and Kaiowá children in Mato Grosso do Sul, Brazil, similarly found a proxy indicator of higher income to be a protective factor against dental caries, likely due to greater access to fluoride and higher quality diets associated with human capital, selfcare, and social inclusion [11]. In addition to linking higher household socioeconomic status with fewer dental caries, our findings also elucidate a robust set of interrelated contributing factors pointing to the possible importance of relative socioeconomic inequality over the life course.

Although the magnitudes of differences observed between village groups in cultivated, forest, and industrialized foods scores were not large, they made important contributions to the overall interaction of household indicators and socioeconomic dynamics among households and village groups, and ultimately to oral health. Importantly, per capita income and durable goods were not linked to dental caries directly, but rather predicted better oral health indirectly via household emphasis on cultivated foods. Thus, in this study population, socioeconomic condition contributed to oral health determination by means of its intermediary position between village groups and household food economies.

This complex scenario suggests that relative household socioeconomic differentiation and historically derived differences between village groups, such as economic profiles and geographical location, play important roles in determining the kinds of foods households acquire [19]. These groups’ divergent histories during recent years and decades likely entailed contrasting trajectories of life experiences involving market insertion and food acquisition with apparent implications for oral health determination. In this general sense, our findings involving household socioeconomic status and food economies may be interpreted as congruent with accumulated and critical-period effects models [43].

The finding that greater access to cultivated foods directly predicted fewer dental caries was not expected considering prevalent understandings of oral health consequences of historical dietary transitions from collecting, hunting, and fishing to cultivation of high-starch foods. As occurred in many global populations during the Agricultural Revolution and more recently in numerous Indigenous groups in Central Brazil and Amazonia [34,36,44,45], such changes are usually considered to favor increased caries. Similarly, the observed absence of a significant association between industrialized foods scores and dental caries may appear inconsistent with literature showing that increased market engagement by Indigenous peoples exposes them to increased consumption of cariogenic foods and drinks [36,39]. The results of our model suggest an alternative explanation for the link between emphasis on cultivated foods and better oral health among the Xavante: household cultivated foods scores contributed to the determination of dental caries as part of an interrelated complex of household socioeconomic dynamics rather than as a narrow indicator of cariogenic dietary conditions.

The Xavante peoples’ experience of market insertion and subsistence change since the 1940s involved the emergence of new forms of internal socioeconomic differentiation through unequal access to social benefits and other sources of income [14,18,19]. As these groups dispersed within the Pimentel Barbosa and Wedezé reserves over the last four decades, differentiation also occurred in their proximities to landscape resources and urban areas, as well as their engagement with external social and economic spheres. As compared to village group 1, most villages in groups 2 and 3 were closer to a small town and federal highway [19]. Villages in groups 2 and 3 were also situated in more environmentally degraded landscapes due to prior occupation and deforestation by large ranches in the 1970s [29]. These circumstances may have contributed to the transformation of gardening, which was historically less important to Xavante household food economies than gathering, hunting, and fishing, into an alternative economic strategy associated with increased market insertion, with positive repercussions for oral health, especially in village groups 2 and 3.

### Oral health services and selfcare

About three decades after Neel and Salzano’s seminal study, Arantes began a long-term research program addressing dental caries epidemiology [30,31] and from 1999 to 2009 developed a preventive and clinical oral health program to residents of Pimentel Barbosa and Etênhiritipá villages, located in the Pimentel Barbosa Indigenous Reserve. Our findings show this program was directly linked to fewer dental caries. The private oral health program’s favorable results in village group 1 has been attributed to immediate benefits of free access to basic oral healthcare and enduring impacts of preventive care and educational interventions [10]. One of the program’s most effective preventive measures was topical fluoride therapy, considering that village water sources were not fluoridated. The observed indirect link from presence of the oral health program to fewer dental caries via toothbrushing suggests that the program’s emphasis on routine educational activities for school children and providing free access to toothbrushes and fluoride toothpaste were also effective.

Compared to several other Indigenous ethnic groups in Brazil, this Xavante population had considerably lower levels of oral healthcare use. The mean proportion of individuals in all Xavante village groups who reported never having visited a dentist was 47.2%, whereas these values were reported to be just 32.8%, 32.6%, and 24.3% among Terena, Kaiwoá, and Guarani populations, respectively, in 2013 [11]. Use of dental services in our study population was uneven between villages, with residents of the villages in group 1 that benefitted from the oral health program reporting significantly more use than the other two village groups.

Results of the parsimonious model showed access to the oral health program directly predicted more frequent toothbrushing and the village group sequence indirectly predicted lower toothbrushing frequency via less use of dental services, suggesting a pertinent factor in low use was lack of access to services. The finding that use of dental services indirectly predicted fewer dental caries via toothbrushing suggests that access to healthcare also stimulated improved selfcare. The observed interrelationships between the oral health program and use of dental services along with links between village group, socioeconomic status, and household food economies support assertions that services provisioning should be understood to contribute to oral health determination and solutions within broad social and lifestyle frames [46,47].

Whereas relatively immediate or proximal contributions to health determination are often attributed to selfcare, our findings suggest oral selfcare among the Xavante was historically derived within the lifetimes of our study participants. Notably, the historical village group sequence directly linked to use of dental services. Also, toothbrushing frequency mediated links to oral health from the sequence of village group divisions (1980s to 2000s), presence of a private oral health program (1999 to 2009), and use of dental services (within an individual’s lifetime). Thus, the contemporary relationship between oral selfcare and dental caries accompanies historical dimensions of access to oral health services and interventions.

### Sex and age

The observed direct links from sex and age to dental caries may be attributed to expected relationships involving biological factors. For example, higher caries prevalence among women has been shown clinically to be affected by hormonal changes during pregnancy that affect oral tissues and reduce resistance to cariogenic conditions [48,49]. Previous studies have argued that the high fertility of Xavante women [18] may increase the frequency and duration of these alterations [18,41]. Similarly, the expected relationship between older age and increased dental caries is largely attributed to accumulation of dental caries through time and reduced salivary gland function in older age [50,51].

Previous research among the Xavante has also attributed the sex differential in dental caries to culturally specific social, economic, and dietary differences associated with gender, along with the biological aspects of reproduction and fertility [31]. The association observed in the present study between female sex and less frequent toothbrushing is congruent with the behavioral aspect of this argument, with males tending to have more favorable oral selfcare practices due to greater access to schooling and engagement with external Brazilian society. Similarly, the finding that being older directly predicted greater use of dental services and indirectly predicted higher toothbrushing frequency suggests the biological tendency for older people to accumulate more dental caries through time appears to be mitigated by selfcare behaviors among our study participants. It is possible that such factors as access to education and life experience contribute to older people using dental services and brushing their teeth more than younger people, with positive repercussions for their oral health.

Our results regarding sex and age may also be interpreted according to theoretical frameworks that address oral health causality in terms of broader historical, social, and political circumstances (causes-of-causes) and thereby avoid overemphasis on individual choice, life-course, and intrinsic cultural-behavioral determinism [47,52]. The observed relationships linking sex and age with dental caries were situated within the same overall current of historical change as the village group sequence and presence of the oral health program. The entire population now residing in the Pimentel Barbosa and Wedezé Indigenous reserves that was the focus of the present study was reduced to about 200 individuals by 1969 due to their unfavorable experience of contact and initial engagement with Brazilian society. It has now grown to approximately 1,500 due to increased fertility and reduced child mortality, partially due to improved access to public health and sanitation services [14,18]. Consequently, the biology of Xavante women’s buccal environments associated with fertility is fundamentally historically determined. Similarly, the accumulation of dental caries over the life course is a relatively recent phenomenon among the Xavante, considering dental caries were essentially non-existent in the early 1960s [22,40] and may be largely attributed to economic and dietary changes associated with increased insertion in Brazilian markets and society.

Since the 1960s, access to schooling increased, societal and political attitudes towards Indigenous people have shifted, and the life experiences of young people have diverged from those of their parents and grandparents. Within the study population, the social meanings of gender difference are in flux as Xavante people engage with and reinterpret Brazilian cultural gender norms [20,53]. Unfortunately, the public healthcare system is inadequately prepared to deal with sociocultural diversity [54], potentially causing reduced use of health services due to communication barriers and perceptions of prejudice, especially among the predominantly monolingual female population in this study. Thus, ostensibly cultural and life course factors such as gender, childhood education, and access to health services are changing through the same historical processes of differentiated territorial and oral care histories that were foundational to our theoretical model. The Xavante case would suggest there is value in Anne-Marie Nybo Andersen’s comment “I would recommend reading the [life-course] articles while listening to Bob Dylan’s The Times They Are a-Changin’: an ultimate reminder of some of the difficulties in life-course research” [55] (p. 541).

Several study limitations should be considered. The cross-sectional nature of the data precludes causal inference between the investigated variables. Variables derived from interview responses (especially socioeconomic status, household food acquisition, health services use, and selfcare) might lack precision as compared to observational variables (such as caries). In addition, our data do not differentiate between use of fluoride and non-fluoride toothpaste.

## Conclusion

Accentuated social and oral health inequities between Indigenous and non-Indigenous populations are observed in most world regions [56]. They often accompany historical transformations of Indigenous economies and living conditions through increased insertion in national societies and markets [56,57] and are partially attributable to such macrodeterminants as social exclusion and institutional racism, limited access to formal education, exclusion from employment opportunities, and lack of access to health services [58]. Marked oral health disparities attributed to social exclusion are similarly observed between Indigenous peoples and their respective national populations in multiple world regions [59]. In Brazil, the exclusion of certain social segments from recent oral health improvements is well substantiated [3,5,6,60]. The few studies that have addressed social determinants of dental caries among Indigenous peoples have shown associations with geographical location, physical characteristics of the house, demographics, per capita income, diet and oral cleanliness patterns, and access to health services [10,18,60].

Our study assessed a diverse set of potential socioeconomic, food acquisition, and healthcare determinant factors according to a hypothetical model grounded in our previous ethnographic and public health research in the Pimentel Barbosa and Wedezé reserves. One of our central questions was how differentiated territorial and oral care histories of Xavante residing in the Pimentel Barbosa and Wedezé reserves contribute to the determination of dental caries considering national and global processes of social exclusion as they are situated and engaged locally. Building on theoretical and analytical frameworks that are inadequately addressed in previous research on oral health among Indigenous peoples, this community study of Xavante residents of Pimentel Barbosa and Wedezé Indigenous reserves, Central Brazil, provides broad insights into how multiple scales and dimensions of historically derived determinants can affect emergent oral health disparities in marginalized ethnic minorities within national contexts of recent improvements in oral health and generalized access to preventive oral health services.

All three historical processes contemplated in our theoretical framework (rapid population growth, major economic change, and improved access to oral health services in recent decades) appear to be involved in the significant interrelated links contributing to dental caries determination. For example, possible biological connections between pregnancy and reduced resistance to cariogenic conditions suggest that the observed link between female sex and increased dental caries may be connected to the recent historical context of high fertility among Xavante women. Also, living in households with more favorable socioeconomic conditions and increased access to food resources was linked to fewer dental caries within a specific historical frame of marked social, political, and environmental transformation within the lifetimes of the study’s participants. Simultaneously, recent changes in local and regional health policies and access to health education are intrinsic to the observed positive associations between dental caries and greater access to oral health services and more frequent oral selfcare.

Some of our results potentially lend support to established theories of health determination. For example, we observed interrelated relationships linking dental caries to different measures of household socioeconomic status, food economies, and oral health services and care that may be interpreted in terms of life course frameworks, including critical-period and accumulated effects [43]. Similarly, the expected findings of more dental caries and less frequent toothbrushing among women and caries accumulation and greater use of dental services among older individuals could be interpreted as lending support to biological and behavioral determination arguments.

These interpretive tools may be useful for understanding some of the specific relationships observed in our results, but a more complete picture of oral health determination within the study population emerges by considering the larger historical frame of territorial circumscription and demographic crisis followed by rapid population increase since the 1970s, which should be considered a cause-of-cause determinant [61] of the village divisions and differentiation contributing to the differences in oral health use, selfcare, and caries status observed in this study. Even the contemporary biology of Xavante women’s and elders’ buccal environments, which conformed to epidemiological expectations, appears to be a recent phenomenon resulting from a specific historical tide of economic and social change produced through national politics over the last half century. Specifically, the Brazilian agenda of internal colonialism and economic growth by means of the settlement and assimilation of Indigenous peoples into an economically productive Brazilian society [14] was a major historical contributor to our results. The specific significant determinant pathways encountered in this study may differ from those present in other Xavante communities and in Indigenous communities pertaining to other ethnic groups with different historical, social, and health conditions. In a more general sense, our findings illustrate that Indigenous peoples’ oral health epidemiology is closely linked to the broad historical tendencies that determine social and economic relationships between minority ethnic groups and national societies.

## Supporting information

S1 Interview Questions Interview questions utilized in statistical analyses, presented in Portuguese (original language) and translated into English. (PDF)
