# Supplementary material for: Human ecological and social determinants of dental caries among the Xavante Indigenous people in Central Brazil
Source: PLoS One. 2018 Dec 19;13(12):e0208312. doi: 10.1371/journal.pone.0208312 (PMC6300322; doi:10.1371/journal.pone.0208312)
Supplement: S1 Interview Questions — (PDF) [file pone.0208312.s001.pdf]

S1 Interview Questions

Portuguese language interview questions utilized in statistical analyses

Perguntas na entrevista domiciliar

1) Com qual frequência os membros de sua casa costumam comer cada alimento de cultivo ou criação ao longo do ano? (respostas: 1–nunca ou raramente; 2–só às vezes ou só em uma época; 3–freqüente-mente ou todo dia; 99–IGN):

    a) Arroz da roça
    b) Milho Xavante
    c) Milho não-Xavante
    d) Abóbora
    e) Feijão Xavante
    f) Feijão não-Xavante
    g) Mandioca/farinha
    h) Batata doce
    i) Raízes e tubérculos cultivados
    j) Melancia
    k) Cana
    l) Banana
    m) Mamão
    n) Frango de criação
    o) Ovos de galinha de criação
    p) Carne de boi de criação

2) Com qual frequência os membros de sua casa costumam comer cada alimento do mato ou rio ao longo do ano? (respostas: 1–nunca ou raramente; 2–só às vezes ou só em uma época; 3–freqüente-mente ou todo dia; 99–IGN):

    a) Frutas do mato
    b) Raízes/tubérculos
    c) Palmitos
    d) Mel
    e) Peixe
    f) Carne de tartaruga, tracajá ou jabuti
    g) Ovos de tartaruga ou tracajá
    h) Aves silvestres
    i) Caça pequena
    j) Caça de grande porte
    k) Formigas

3) Com qual frequência os membros de sua casa costumam comer cada alimento da cidade ao longo do ano? (respostas: 1–nunca ou raramente; 2–só às vezes ou só em uma época; 3–freqüente-mente ou todo dia; 99–IGN):

    a) Arroz comprado
    b) Feijão comprado
    c) Frango congelado
    d) Ovos de frango comprados
    e) Carne de boi ou linguiça
    f) Peixe congelado
    g) Café
    h) Açúcar
    i) Sal ou Arisco
    j) Óleo de cozinha
    k) Extrato de tomate
    l) Cebola ou alho
    m) Macarrão
    n) Trigo ou pão
    o) Bolachas, biscoitos ou salgadinhos
    p) Refrigerante ou suco em pó
    q) Balinhas ou bombons

4) Para cada item, indique a quantidade que os membros da sua casa possuem:

a) Televisão
b) DVD
c) Antena parabólica
d) Fogão a gás
e) Máquina de costura
f) Gravador de fita (pequeno)
g) Som portátil
h) MP3/MP4/etc.
i) Telefone celular
j) Câmera de rolo de filme
k) Câmera digital
l) Câmera de vídeo
m) Computador ou notebook
n) Espingarda
o) Revólver
p) Rede de pesca
q) Moto
r) Automóvel
s) Bicicleta
t) Motor/gerador
u) Caixa de som
v) Geladeira ou freezer

5) Atualmente, quais são os valores das rendas dos membros da sua casa? (incluir salários, aposentadorias, pensões, bolsas família, auxílios, freelance, etc.)

Perguntas na entrevista individual

1) Qual é seu sexo? (respostas: 0–homem; 1–mulher)

2) Qual é sua data de nascimento?

    a) Se não se sabe sua data de nascimento, quantos anos você tem?

3) Você costuma limpar os dentes? (respostas: 0–não; 1–sim; 99–IGN)

    a) SE SIM, com qual frequência? (respostas: 0–às vezes, quando lembro; 1–uma vez ao dia; 2–duas a três vezes ao dia; 99–IGN)

    b) SE SIM, o que você costuma fazer/usar? (respostas: 0–pasta de dente e escova; 1–bochechar com água; 2–outro; 99–IGN)

4) Você já foi atendido pelo dentista alguma vez? (respostas: 0–não; 1–sim; 99–IGN)

    a) SE SIM, quando foi a última vez? (respostas: 0–no último ano; 1–há mais de um ano; 99–IGN)

Interview questions utilized in statistical analyses, translated from original language (Portuguese) to English

Questions from household interviews

1) How frequently do the members of your household usually eat each cultivated or raised food item? (responses: 1–never or rarely; 2–sometimes or seasonally; 3–frequently or daily; 99–unknown or no response):

   a) Planted rice
   b) Xavante maize
   c) Non-Xavante maize
   d) Squash
   e) Xavante beans
   f) Non-Xavante beans
   g) Manioc/manioc meal
   h) Sweet potato
   i) Cultivated roots and tubers
   j) Watermelon
   k) Sugarcane
   l) Banana
   m) Papaya
   n) Raised chicken
   o) Raised chicken eggs
   p) Raised beef

2) How frequently do the members of your household usually eat each cultivated or raised food item from the forest or river? (responses: 1–never or rarely; 2–sometimes or seasonally; 3–frequently or daily; 99–unknown or no response):

   a) Forest fruits
   b) Roots and tubers
   c) Heart of palm
   d) Honey
   e) Fish
   f) Turtle or tortoise meat
   g) Turtle eggs
   h) Wild birds
   i) Small game animals
   j) Large game animals
   k) Ants

3) How frequently do the members of your household usually eat each food item from the city? (responses: 1–never or rarely; 2–sometimes or seasonally; 3–frequently or daily; 99–unknown or no response):

   a) Purchased rice
   b) Purchased beans
   c) Frozen chicken
   d) Purchased chicken eggs
   e) Beef or sausage
   f) Frozen fish
   g) Coffee
   h) Sugar
   i) Salt
   j) Cooking oil
   k) Tomato extract
   l) Onion or garlic
   m) Pasta
   n) Wheat flour or bread
   o) Cookies, crackers, or chips
   p) Soda or powdered juice
   q) Candy

4) For each item, indicate the quantity owned by members of your household:

   a) Television
   b) DVD player
   c) Parabolic antenna
   d) Gas oven
   e) Sewing machine
   f) Tape recorder (small)
   g) Portable music player
   h) MP3 player
   i) Cellular telephone
   j) Film camera
   k) Digital camera
   l) Video camera
   m) Computer or notebook
   n) Shotgun
   o) Revolver
   p) Fishing net
   q) Motorcycle
   r) Automobile
   s) Bicycle
   t) Generator
   u) Loudspeaker
   v) Refrigerator or freezer

5) What are the current income values of members of your household (include wages, pensions, social benefits, freelance income, etc.)?

Questions from individual interviews

5) What is your sex? (responses: 0–male; 1–female)

6) What is your date of birth?

   a) If you do not know your birthdate, how old are you?

7) How often do you usually clean your teeth? (responses: 0–no; 1–yes; 99–unknown or no response)

   a) IF YES, how frequently? (responses: 0–sometimes, when I remember, 1–once per day; 2–twice or three times per day; 99–unknown or no response)

   b) IF YES, what do you usually do/use? (responses: 0–toothpaste and brush; 1– rinse with water; 2–other; 99–unknown or no response)

8) Have you been seen by a dentist at least once? (responses: 0–no; 1–yes; 99–unknown or no response)

   a) IF YES, when was the last time? (responses: 0–during the past year; 1–more than one year ago; 99–unknown or no response)
